# Interaction between audiology and genetics in the study of a family: the complexity of molecular diagnosis and genetic counseling

**DOI:** 10.1016/S1808-8694(15)31379-3

**Published:** 2015-10-17

**Authors:** Flavia Maria Rodrigues Hoffmann, Patrícia Fernandes Rodrigues, Teresa Maria Momensohn dos Santos, Edi Lucia Sartorato, Andréa Trevas Maciel-Guerra, Carla Gentile Matas, Vanessa Cristine Sousa de Moraes

**Affiliations:** 1 Specialist in audiology at Instituto de Estudos Avançados da Audição, IEAA, Brazil. Speech and Hearing Therapist; 2 PhD in Human Communication Disorders, Speech and Hearing Therapy at Universidade Federal de São Paulo, UNIFESP, Brazil. Speech and Hearing Therapist, Professor in the specialization program on Audiology at Instituto de Estudos da Audição - IEAA; 3 PhD in Human Communication Disorders, Speech and Hearing Therapy at Universidade Federal de São Paulo, UNIFESP, Brazil. Speech and Hearing Therapist, Full Professor at PUC SP, Director at Instituto de Estudos Avançados da Audição - IEAA; 4 PhD in Genetics and Molecular Biology at Universidade Estadual de Campinas, UNICAMP, Brazil. Researcher at Centro de Biologia Molecular e Engenharia Genética da UNICAMP, Associate Professor of Medical Genetics at UNICAMP; 5 PhD in Genetics and Molecular Biology at Universidade Estadual de Campinas, UNICAMP, Brazil. MD, Geneticist, Full Professor at the Medical Genetics Department at Faculdade de Ciências Médicas da UNICAMP; 6 PhD in Human Communication Disorders, Speech and Hearing Therapy at Universidade Federal de São Paulo, UNIFESP, Brasil. Adjunct Professor in the Speech and Hearing Therapy program at the Department of Physiotherapy, Speech and Hearing Therapy, and Occupational Therapy at Faculdade de Medicina da USP; 7 Biologist. Scholarship holder on Technical Training at FAPESP’s Centro de Biologia Molecular e Engenharia Genética. IEAA - Instituto de Estudos Avançados da Audição

**Keywords:** counseling, hearing loss, genetics, mutation

## Abstract

Hearing loss is a multifaceted condition with many etiologies, among which genetic mutation is. Therefore, it is important to connect audiological investigation to etiological diagnosis.

**Aim:**

this study aims to establish the audiological and genetic profiles of three non-syndromic children with sensorineural hearing loss.

**Materials and method:**

three brothers aged 3, 5 and 16 were enrolled in this study. They were submitted to behavioral and electrophysiological hearing tests and molecular studies.

**Results:**

the hearing tests showed moderate to moderately severe bilateral symmetric sensorineural hearing loss and an accentuated descending slope. Transient and Distortion Product Otoacoustic emissions were absent in the two younger children. ABR showed a bilateral moderately severe to severe sensorineural hearing loss. P300 showed bilateral normal latencies in the older brother. Molecular tests showed that the two younger children were heterozygote for mutation 35delG on gene GJB2.

**Conclusion:**

The combination of speech and hearing tests and genetic analysis allows for the etiologic diagnosis of seemingly similar hearing loss cases, which however display different genetic backgrounds. Molecular studies must be comprehensive enough to avoid precipitated diagnosis which may impair genetic counseling.

## INTRODUCTION

Hearing loss (HL) is the most frequently diagnosed sensorial deficit. This condition impairs the exertion of communication skills^1^ and introduces a series of social, psychical, and educational hurdles to those affected by it. When diagnosed by the first year of life, medical and speech and hearing therapy approaches can be devised to critically act on a still maturing and more plastic central nervous system, thus improving the global prognostic possibilities of the involved child^2^.

Behavioral and electrophysiological tests are available to assist with the audiological diagnosis, and may be used as part of the assessment procedure^3^, ^4^, ^5^, ^6^, ^7^, ^8^, ^9^, ^10^, ^11^. Test applicability is based on patient maturation and development status.

Congenital sensorineural hearing loss may or not have a genetic background. Within the genetically-originated cases, there are those in which HL is part of a syndrome (30%) and those in which it is an isolated condition - non-syndromic sensorineural HL (70%)^12^, ^13^, ^14^. Over 100 genes are potentially involved in non-syndromic prelingual HL^14^, which originates in 85% of the cases from recessive autosomal inheritance, in 12% to 14% from dominant autosomal inheritance, and in 1% to 3% is bound to chromosome X^15^, ^16^.

Within the cases of non-syndromic recessive autosomal inheritance HL are the ones occurring from mutations on gene GJB2, in charge of encoding protein conexin 26. One of these mutations, 35delG, accounts for about 70% of the mutant alleles in European and North-American Caucasians, with 2.3% to 4% heterozygotes^17^, ^18^, ^19^, ^20^. In Brazil, the heterozygote frequency of mutation 35delG is estimated at 1:7421. Other genetic alterations found in non-syndromic HL patients are deletions on the gene connected to conexin 30 (GJB6-Cx30), del (GJB6 - D13S1830) and del (GJB6 - D13S1854), as well as mutation A1555G on mitochondrial gene 12SrRNA^22^, ^23^, ^24^.

In developing countries environmental factors such as congenital infections and perinatal disorders are still quite common^25^. However, as health care to mothers and infants improves, the relative frequency of genetic-origin cases progressively increases^26^.

In cases of genetically-acquired hearing loss, additionally to early auditory stimulation and use of hearing aids, genetic counseling must also be considered to allow individuals and their families to make well-informed decisions on procreation^27^. Gene therapy is still being researched, and cannot be applied in every situation^28^.

Genetic counseling for cases of inherited non-syndromic HL relies heavily on accurate diagnosis of the inheritance mechanism. The introduction in Brazil of tests to check for mutation 35delG on gene GJB2, among other exams required to clarify the etiology of the condition has been fundamental for effective genetic counseling. Indeed, its detection is highly relevant in tracking the origins of HL, thus reinforcing the relevance of having comprehensive molecular to avoid precipitated diagnosis and unnecessary stress for the families.

This paper aims to report on the audiological profiles and genetic test results of a family from the municipality of São Paulo, emphasizing the genetic heterogeneity of HL patients and the complexity of genetic counseling.

## MATERIALS AND METHOD

This study was approved by the Research Ethics Committee at CEFAC under permit 172/05.

**Figure 1:**
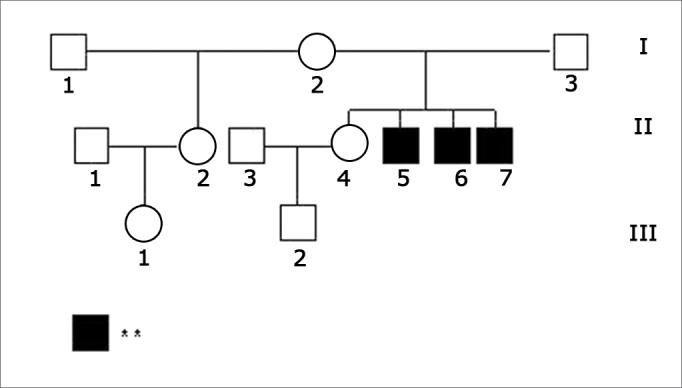
Inheritance patterns of the family analyzed in this study. - Legend: ** Hearing loss.

The index-case (II.5, Fig. 1), male, HL patient, was seen for the first time when he was 16 at the Clinical Audiology ward at IEAA in November of 2004, after his speech and hearing therapist asked him to undergo audiological examination. He was the second of four children of a non-consanguineous couple. The family consisted of a 41-year-old mother (I.2), a 40-year-old father (I.3), an older 17-year-old sister (II.4) with hearing sensitivity within normal standards who had a one-year-old son (III.2), and two younger brothers of five (II.6) and three years of age (II.7) respectively, both with hearing loss. There was also an older, 21-year-old half-sister (II.2) from the mother’s side also with normal hearing, with a one-year-old daughter (III.1). The three affected siblings had prelingual non-progressive HL.

### Audiological examination

Audiological examination included both behavioral and electrophysiological tests, conducted based on the needs of each participant considering their ages and presence of hearing impairment.

Tone audiometry (TA), speech audiometry (SA), impedance tests (IT), acoustic reflex measurements (ARM), distortion product otoacoustic emission (DPOAE) tests, and transient-evoked otoacoustic emission (TEOAE) tests were conducted at the IEAA.

**Chart 1:**
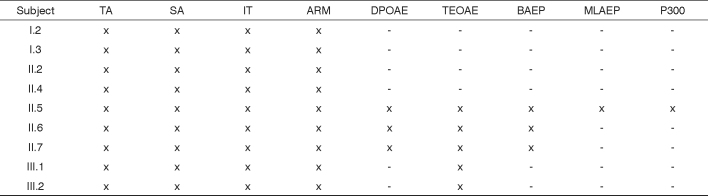
Audiological test results per subject.

Brainstem auditory evoked potential (BAEP), middle-latency auditory evoked potential (MLAEP), and P300 tests were conducted at the CDP at FMUSP. Chart 1 shows the audiological findings for all family members.

### Molecular studies

Genetic tests were carried out at the Center for Molecular Biology and Genetic Engineering at UNICAMP. Presence of mutation 35delG on gene GJB2 was investigated using *AS-PCR (allele-specific polymerase chain reaction) to discriminate normal from mutant alleles, thus telling normal homozygotes from 35delG homozygotes and mutated heterozygotes; this gene was also completely sequenced.

Deletions involving the conexin 30 gene (GJB6-Cx30) were also analyzed - del (GJB6 - D13S1830) and del (GJB6 - D13S1854) - using multiplex PCR; mutation A1555G on mitochondrial gene 12SrRNApor was searched through restriction analysis with digestion of enzyme Bsm AI.

## RESULTS

Audiological evaluation of the index-case (II.5) and siblings (II.6 and II.7) showed an audiometric curve with sensorineural bilateral symmetric hearing loss, with an accentuated descending configuration. Individuals II.5 and II.7 had moderately severe bilateral hearing impairment and II.6 had moderate hearing loss in the right ear and moderately severe HL on the left ear. The parents (I.2 and I.3), sister (II.4), half-sister (II.2) and nephews (III.1 and III.2) had normal hearing thresholds. DPOAEs were absent on II.6 and TEOAEs were absent on subject II.7.

BEAP test results were consistent with severe bilateral hearing loss for clicks on subjects II.5 and II.7 and moderately severe on II.6. P300 showed latencies within normal range bilaterally for subject II.5.

**Figure 2:**
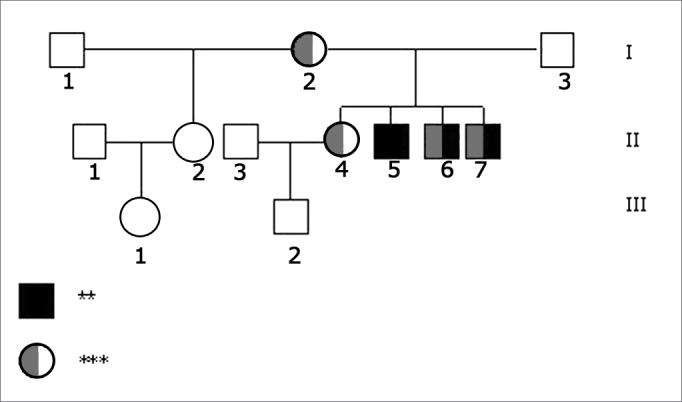
Family inheritance patterns showing genetic test results. - Legend: ** Hearing loss *** Heterozygote 35delG N - Normal allele NR - Not realized.

In terms of molecular tests, none of the analyzed individuals had deletions in the conexin 30 gene or mutations on gene 12SrRNA. Mutation 35delG in heterozygosis was found in the index-case mother’s (I.2) gene GJB2, in the older sister (II.4) with normal hearing and in the two siblings with hearing impairment (II.6 and II.7). The father (I.3), the index-case (II.5) and the half-sister’s daughter (III.1) are normal homozygotes, and thus did not have mutation 35delG; unfortunately it was not possible to perform molecular tests on the half-sister (II.2) and the nephew (III.2). Molecular findings can be seen in the inheritance patterns shown in Figure 2.

## DISCUSSION

Hearing impairment introduces a series of restrictions and difficulties into the lives of those affected by it^1^. Early diagnosis is crucial in offering patients the best intervention possible^2^, as is an effective interaction between speech and hearing therapy and genetics.

The thorough audiological examination performed with the core of the family (parents and siblings) added to the tests done with the half-sister and the nephews revealed that the three siblings had practically the same kind of hearing loss, and that their parents, sister, half-sister and nephews had normal hearing^3^, ^4^, ^5^, ^6^, ^7^, ^8^, ^9^, ^10^, ^11^.

The study also showed that the identified hearing impairment was of the sensorineural prelingual non-syndromic type^12^, ^13^, ^14^.

Many are the possible inheritance mechanisms^15^, ^16^ at play, the most likely being the recessive autosomal, in which the normal parents do not present the phenotype, are heterozygote carriers, and have the disorder manifesting itself in their offspring; another possibility is a recessive inheritance pattern connected to chromosome X, with a normal phenotype heterozygote carrier mother with the disorder affecting only male individuals.

It is still possible to consider dominant autosomal inheritance, with incomplete penetrance in one of the parents or consequent to germinative tissue mutation in one of them^15^, ^16^, and maternal or mitochondrial inheritance^24^, with preferential clinical manifestation in males, as is the case in Leber’s optic neuropathy.

The limitations related to the diagnosis of patients with non-syndromic sensorineural hearing loss through molecular tests pose an interesting challenge. If the genetic tests had been limited to one of the siblings carrying mutation 35delG (II.6 and II.7) and one of the parents, we would have looked at explaining the presence of the mutation in heterozygosis in individuals with hearing loss. Indeed, one of the biggest difficulties in offering genetic counseling to individuals carrying mutations in the conexin 26 gene - including mutation 35delG - is the fact that in 10% to 40% of the cases these mutations are detected only in one of the alleles, but still result in hearing loss. In some cases these mutations segregate with deletions in gene GJB6, i.e., the individual is heterozygote for mutations in gene GJB2 and in the other allele he or she presents one of the two deletions involving gene GJB6^22^, ^23^. In the family we analyzed, however, no such deletions were found. In monoallelic cases with mutation in gene GJB2, it is not possible to exclude interactions between mutations in the conexin 26 gene and in other genes related to hearing loss in a digenic inheritance pattern. Another possibility to explain this situation is the presence of modifier genes with dominant negative effect upon the normal allele, which in this case would depend on the genetic background of each individual.

Still in reference to the scenario in which subject II.5 did not undergo molecular testing, any geneticist would see as the best possibility a situation in which the interaction occurred in the other allele is not explained, and thus mutation 35delG would be connected to the hearing loss phenotype presented by the family.

Therefore, recessive inheritance connected to chromosome X could be excluded and thus the risk of the sister (II.4) and the half-sister (II.2) having male children with hearing loss would not be considered, as nor would mitochondrial inheritance, thus also discarding the risk for the offspring of the sister and that of the half-sister.

However, as the core of the family was entirely studied, we could verify that the hearing loss phenotype is independently segregated from genotype 35delG. Indeed, there were two individuals carrying the mutation with yet normal hearing (I.2 and II.4) and one with hearing loss but without mutation 35delG (II.5).

Mutation 35delG can thus be considered to be a casual finding, given the fact that this mutation in heterozygosis is quite common within the unaffected, getting to as much as 4% in some populations^17^, ^18^, ^19^, ^20^, ^21^. All possibilities initially considered to explain the inheritance mechanism remain valid, thus making it impossible to accurately calculate the risk of recurrence of the impairment in the family and thus compromising any genetic counseling effort.

## CONCLUSION

This paper stresses the importance of multidisciplinary diagnosis and the need of establishing closer ties between speech and hearing therapy and genetics to enhance the etiologic diagnosis and genetic counseling offered to hearing loss cases.
